# Current State of Non-wearable Sensor Technologies for Monitoring Activity Patterns to Detect Symptoms of Mild Cognitive Impairment to Alzheimer's Disease

**DOI:** 10.1155/2021/2679398

**Published:** 2021-02-10

**Authors:** Rajaram Narasimhan, Muthukumaran G., Charles McGlade

**Affiliations:** ^1^Centre for Sensors and Process Control (CENSE), Hindustan Institute of Technology and Science, 1, Rajiv Gandhi Salai (OMR), Chennai 603 103, India; ^2^Ridgeline Management Company (Senior Living), 1914 Willamette Falls Dr. #230, West Linn, OR 97068, USA

## Abstract

Mild cognitive impairment (MCI) could be a transitory stage to Alzheimer's disease (AD) and underlines the importance of early detection of this stage. In MCI stage, though the older adults are not completely dependent on others for day-to-day tasks, mild impairments are seen in memory, attention, etc., subtly affecting their daily activities/routines. Smart sensing technologies, such as wearable and non-wearable sensors, coupled with advanced predictive modeling techniques enable daily activities/routines based early detection of MCI symptoms. Non-wearable sensors are less intrusive and can monitor activities at naturalistic environment with no interference to an individual's daily routines. This review seeks to answer the following questions: (1) What is the evidence for use of non-wearable sensor technologies in early detection of MCI/AD utilizing daily activity data in an unobtrusive manner? (2) How are the machine learning methods being employed in analyzing activity data in this early detection approach? A systematic search was conducted in databases such as IEEE Explorer, PubMed, Science Direct, and Google Scholar for the papers published from inception till March 2019. All studies that fulfilled the following criteria were examined: a research goal of detecting/predicting MCI/AD, daily activities data to detect MCI/AD, noninvasive/non-wearable sensors for monitoring activity patterns, and machine learning techniques to create the prediction models. Out of 2165 papers retrieved, 12 papers were eligible for inclusion in this review. This review found a diverse selection of aspects such as sensors, activity domains/features, activity recognition methods, and abnormality detection methods. There is no conclusive evidence on superiority of one or more of these aspects over the others, especially on the activity feature that would be the best indicator of cognitive decline. Though all these studies demonstrate technological developments in this field, they all suggest it is far in the future it becomes an effective diagnostic tool in real-life clinical practice.

## 1. Introduction

In a global study and report published by Alzheimer's disease International (ADI) [1], it is estimated that dementia affects 50 million people, costing the global economy over US$1 trillion. Someone in the world develops dementia every 3 seconds. It is estimated that the number will almost double every 20 years, reaching 75 million in 2030 and 131.5 million in 2050. The implications of this suggest devastating impacts on healthcare costs, quality of life of patients, and their caregivers. Dementia is a neuro-degenerative condition in which there is deterioration in memory, thinking, behavior, and the ability to perform everyday activities. Although dementia mainly affects older adults, it is not a normal part of ageing. Although there is no treatment currently available to cure dementia, the cause and prevention of this are undergoing intense research efforts. Several studies and analyses demonstrate that treating this condition at its earliest stage will be more effective in terms of social and fiscal outcomes [2, 3].

According to WHO [4], Alzheimer's disease (AD) is the most common form of dementia and may contribute to 60–70% of cases. Since the progression of this neuro-degeneration such as AD can span as long as 30 years, it is important to detect this condition as early as possible. Studies find that certain interventions/treatments, when applied early, can delay and minimize the symptoms of AD in cognitive and behavioral domain [5]. Development of AD is understood to occur in three stages. The first is the prodromal or preclinical stage where certain physiological changes start evolving (especially microscopic changes in brain such as destruction/damage of nerve cells), but individuals present no noticeable symptom making it difficult to distinguish this stage from normal cognitive health. The second state is mild cognitive impairment (MCI) where certain symptoms associated with thinking begin to become noticeable. In this stage, though the older adults are not completely dependent on others for day-to-day tasks, mild impairments are seen in memory, attention, etc., subtly affecting their daily activities. However, MCI does not always lead to dementia. The third, or final stage, is Alzheimer's dementia where cognitive and behavioral symptoms are already evident, and day-to-day function is affected [6]. The third stage itself is often classified into 3 substages: early, mid, and late (although not discrete). In the early stage, day-to-day function is not severely affected; in mid stage, individuals may experience deterioration in memory, problems in solving daily tasks, difficulties in performing every day activities, issues with vision, and difficulties in communication including vocabulary loss; in late stage, individuals become more and more unresponsive and dependent on others even for basic daily activities/personal care.

Several conventional assessment methods (clinical, neuropsychological) exist to evaluate psychological, cognitive, and behavioral symptoms through self-reporting, informant reporting questionnaires, and clinical assessments, typically administered by qualified professionals. Some examples of these tests include for cognitive abilities—Mini Mental State Examination (MMSE), Digit Cancelation Test, Repeatable Battery for the Assessment of Neuropsychological Status (RBANS), Prospective and Retrospective Memory Questionnaire (PRMQ) [7–10]; for mobility testing—TUG, Arm Curl [11, 12]; and for depression assessment—GDS [13]. Often, by the time family members of older adults notice these symptoms and bring them for evaluations, the AD condition may have already progressed resulting in delayed diagnosis. There are certain shortcomings with conventional assessment methods such as they consume lots of time and manual effort, provide point in time observation, necessitate periodic evaluation, do not monitor routine of older adults, at times include biased reporting, and may not give a complete picture of the older adult's functional performance.

MCI is the stage where changes may be noticeable in the performance of daily activities if carefully monitored and could be a transitory stage to a more advanced condition. As a result, research work is focusing on detecting MCI at an early stage so that appropriate interventions can be given to maintain independent living. As discussed above, at MCI stage, older adults experience moderate difficulties in daily routines and activities. Behavioral changes like sleep disturbance, difficulty in walking, inability to complete tasks, etc., can be detected by carefully monitoring the existence of anomalous patterns in daily activities. Daily activities can include basic activities of daily living (ADL) (e.g., bathing, eating, and walking), instrumental ADL (e.g., cooking and using the telephone), and other activities such as sleeping. Several studies suggest that daily activities are appropriate indicators for functional measures to detect MCI at an early stage [14–16].

Advancements in smart sensing technologies have provided plenty of opportunities for researchers to explore possibilities of detecting cognition changes early in older adults. Several studies utilized wearable and non-wearable sensors to monitor activities of older adults and detect behavioral changes. These studies [17, 18] demonstrated that early detection of functional impairments was possible in smart environments by means of continuous monitoring. Wearable sensors have the advantage of higher localization accuracy and tracking; however, they are more intrusive in nature. Also, wearable sensor-based monitoring demands older adults with varying degree of cognitive levels, to remember wearing the devices as well as charge the devices to electricity quite often. On the other hand, non-wearable sensors are less intrusive and can monitor activities at real-life, naturalistic environment without causing any interference to an individual's daily routines. Some examples of non-wearable sensors include motion sensor, door contact sensor, pressure sensor, temperature sensor, and bed mat. Previous research work [19, 20] demonstrated the utility of non-wearable sensing technologies in monitoring older adults' activities unobtrusively and detecting any cognitive decline. Since AD is a degeneration that progresses over time, it is argued that the best indicators of cognitive decline may not necessarily be detected based on one's performance at any single point in time, but rather by monitoring the trend over time and the variability of change in a duration [21]. Since non-wearable sensing technologies enable continuous monitoring of older adults' activities and recognizing the activity trends over time, there is an increased focus in this research area to leverage unobtrusive monitoring in real-life, naturalistic environment. The broad spectra of non-wearable sensors and associated technologies present lots of scope for researchers to select from multitude of sensors, determine optimal sensor topology, and employ varied techniques to extract/recognize activity patterns. Machine learning (a subfield of artificial intelligence) based models have been extensively used in recent research studies to predict the behavioral/cognitive abnormalities utilizing sensor-based activities data. Despite these advantages, there are no established common standards governing sensor selection, activity recognition, and anomaly detection. However, this is an emerging novel research area, and several studies explore to bring advantages of non-wearables based smart sensing in improving quality of life. In this review, we examine current situation of this research area to answer the following questions: (1) What is the evidence for use of non-wearable sensor technologies in early detection of MCI or Alzheimer's disease utilizing daily activity data in an unobtrusive manner? (2) How are the machine learning methods being employed in analyzing activity data in this early detection approach?

## 2. Methodology of Literature Review

We used databases such as IEEE Explorer, PubMed, Science Direct, and Google Scholar to search the relevant articles of our interest. The completed search material encompassed a timeline extending through early March 2019. As a first step, identification of articles was performed by searching abovementioned databases. Our search strategy, in each database, included a combination of key terms with AND, OR logical operators. Predominantly, our search strategy consisted of the terms such as “Smart Home,” “Elders,” “Cognitive Impairment,” “Sensor,” “ADL,” “Prediction,” and “Machine Learning.” Intersection of these terms clearly represents the subject of our interest. Also, our search strategy was restricted to articles in English language. A sample search strategy in IEEE explorer is given below.

((“prediction” OR “monitoring” OR “machine learning” OR “machine learning” OR “supervised learning” OR “unsupervised learning” OR “supervised learning” OR “unsupervised learning” OR “cognitive assessment” OR “detection” OR “predicting” OR “identification” OR “artificial intelligence” OR “support vector machine” OR “artificial intelligence” OR “support vector machine”) AND (“sensor” OR “IoT” OR “sensor data” OR “IoT data” OR “unobtrusive” OR “device” OR “wearable” OR “telemetry”) AND (“smart home” OR “home” OR “activity aware” OR “indoor” OR “house” OR “elder care home” OR “elder care home” OR “home for aged” OR “apartment”) AND (“dementia” OR “cognitive” OR “cognitive impairment” OR “mild cognitive impairment” OR “Alzheimer” OR “MCI” OR “cognitive health” OR “age related disorder” OR “AD” OR “ageing” OR “cognitive deficit” OR “functional deficit” OR “demented” OR “cognitive defect” OR “cognitive decline”) AND (“Activities of daily living” OR “ADL” OR “functional measure” OR “behavior” OR “daily task” OR “activity performance” OR “behavioral feature” OR “activity recognition” OR “functional performance” OR “behavior pattern” OR “Activities of daily living” OR “ADL”) AND (“senior” OR “elderly” OR “elders” OR “resident” OR “older” OR “older adult” OR “older person” OR “independent ageing” OR “graceful ageing” OR “independent living”)) Alzheimer OR dementia

**Figure 1:**
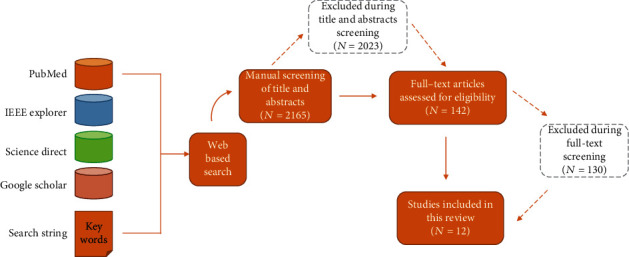
Workflow diagram showing the search and screening method followed.

Pictorial representation of search methodology followed is shown in Figure 1.

As a second step, screening of these articles was done. Screening step included (a) going through the titles and abstract and (b) include or exclude the articles based on the following predetermined criteria:

To be qualified for further review, a research/study:

- Had the research goal of detecting/predicting MCI or AD
- Utilized daily activities dataset as the basis for detection of MCI
- Deployed noninvasive/non-wearable sensors/devices for monitoring activity patterns of older adults
- Included machine learning algorithms/techniques to create the prediction models

Articles with one or more of the below aspects were excluded for further review:

- Goal was to monitor older adults' health condition rather than detection/prediction of cognitive impairment (e.g., fall detection)
- Utilized only intrusive sensors such as video camera or wearables such as accelerometers
- Utilized non-ADL-based approach to detect cognitive impairment or neuro-degeneration (e.g., use of mobile games)

Initial search resulted in 2165 articles. Based on titles/abstracts screening, 142 articles were selected for full-text screening. In the last step of eligibility and finalization, full-text screening of 142 articles was performed, and 12 articles were selected for final review. Main exclusion criteria during eligibility and finalization step were as follows: article being not a research study, duplicate article, insufficient clarity in research method, or insufficient clarity in findings and interpretation.

## 3. Results

Upon searching four electronic databases, we were able to retrieve 2165 English language papers. After screening and review, 12 papers were eligible for inclusion in this review (see Tables 1 and 2) [22–33]. These 12 studies were designed as either longitudinal or cross-sectional, and activities of older adults were monitored through sensors at either their home (regular dwelling unit) or a smart home test bed. While, in longitudinal studies, older adults are monitored continuously using smart sensors, in cross-sectional studies, older adults are asked to perform scripted tasks to assess their functional performance. Study sample size ranged from 1 to 179 participants, and mean age ranged from 60 to 85. There was a wide range of study (or data collection) duration, from 1 hour to 3 years. Number of non-wearable sensors installed at the smart home or smart test bed ranged from 2 to 67. These 12 studies focused on monitoring varied activities (basic ADL, instrumental ADL) and other daily routines such as sleeping and resting, which is in line the with scope of this review.

The nine out of twelve studies [22, 25–27, 29–33] utilized public datasets for their analysis and modeling, and the remaining three studies [23, 24, 28] deployed their own sensors to acquire the activity data. Among public datasets used, CASAS Smart home data (Center for Advanced Studies in Adaptive System–Washington State University) was used by seven studies [22, 27, 29–33], and ORCATECH smart home data (Oregon Center for Aging and Technology at Oregon Health and Science University) was used by two studies [25, 26].

As noted earlier, AD is a degeneration that progresses over time, and it is important to understand the temporal or sequential nature of this disease. Hence, we summarized and classified these 12 studies into two groups depending on whether they considered progressive nature of this disease and performed their sensor data analysis and prediction accordingly. These two groups are, namely, (1) studies that considered progressive nature of degeneration and (2) studies that did not consider progressive nature of degeneration.  Table 1 provides the general characteristics of studies in group 1 [22–27], and Table 2 provides the general characteristics of studies in group 2 [28–33].

In the first group, all these studies followed longitudinal design and adopted different approaches to understand the temporal nature of the progression. One approach adopted was to compute time series statistic features from sensor captured activity data using a sliding time-window method and recognize the behavioral changes over the time [22]. Construction of an activity trend/profile for a subject from sensor activity data was also another approach adopted [23], and this trend/profile indicated the behavioral changes over time. In another approach, all the activities recognized from sensor data on a day per every subject against the same subject's data from previous day to detect the changes and thus recognized the changes that evolved over time [24]. In another approach, based on activity data, behavior models were created which included parameters computed using sliding time-window method and represented the changes evolved over time [25–27].

In the second group, mix of longitudinal [29–31] and cross-sectional [28, 32, 33] studies can be seen. Despite the longitudinal studies in this group collected the activity data over a continuous period, activity/behavior changes happened over the time (temporal nature) were not considered in modeling and analysis. In cross-sectional studies, participants were asked to perform scripted tasks once, and the corresponding activity features were derived for modeling and analysis.

## 4. Discussions

Through our literature search, we finalized 12 papers for this review, and none of these papers was published before year 2013. Not only this shows novelty of the subject of this review but also explains the research in this area is still at emerging stage. These studies illustrated the suitability of non-wearable sensor networks for clinical practice that these sensors were effective in detecting anomalous activity patterns and thus detection of cognitive decline.

The first aim of this review is to provide an overview of the use of non-wearable sensors in early detection of MCI/AD utilizing daily activity data in an unobtrusive manner. We reviewed 12 studies that included a variety of non-wearable sensors with the count ranged from 2 to 67 and a variety of daily activities/routines monitored by these sensors (Table 3). From a single activity to combination of multiple activities were monitored using these sensors. Movement activity domain was the predominant one included in all these studies to detect cognitive/functional decline. Movement domain included mobility of older adults within/outside their residence or movement pattern/trajectory of older adults performing certain activities. Domestic life area was the second most domain included after movement domain among the studies reviewed. Domestic life area included predominantly cooking activity in addition to general housekeeping. Hence, these indicators (movement and domestic life area) of cognitive/functional decline are appropriate choices from technology and clinical perspective.

Few studies [23, 25, 27, 31] considered only one activity domain for monitoring, designed the models accordingly to predict the cognitive decline, and obtained better model performance/outcomes. On the other hand, few studies [27, 31, 32] were able to demonstrate that the use of a single sensor type would be enough in predicting the cognitive/functional decline as opposed to multiple sensor types and further showed better prediction results (except for one study [27] where results were not specified). Choice of sensors and placement/layout of these sensors are so crucial in monitoring systems that they are easily generalizable as well as reproducible in any household set up. Of ten studies reviewed, Schinle et al. [23] utilized only two sensors (1 motion sensor and 1 door contact sensor) in their experiments and were able to detect abnormality with an accuracy as high as 92.3%. This study thus suggests an inexpensive set up for monitoring and appears to be highly generalizable for any household layout. Li et al. [32] and Gochoo et al. [31] derived travel patterns or trajectories from motion sensor data and detected anomalies in participants' motion patterns. Though these studies utilized several sensors, the methodology followed to detect abnormalities appears to be generalizable to any smart home set up. Other studies [22, 29, 30] used several sensors (as high as 38) for monitoring activities and that lead to the question of cost effectiveness and translating complex sensor arrangements to real life situations.

In addition to sensor captured activity data, few studies [22, 28, 32, 33] included nonsensor data such as neuropsychological assessment scores and activity performance scores in their modeling and analysis. These studies found a statistically significant correlation between these two classes of data and defined methods to detect cognitive decline. Though the nonsensor data points provided more contextual features to the prediction models, prediction outcomes of these studies did not differentiate significantly from the studies which utilized only sensor captured data. This raises a question of applicability as well as viability of activity performance scoring in a real-life home monitoring scenario.

Variety of approaches was adopted in computing activity features from raw sensor data and utilizing them in prediction analysis. Given the heterogeneity of the activity features analysis in the studies reviewed, we define two classes of analysis to compare the outcomes, namely, coarse-grained feature analysis and fine-grained feature analysis. In coarse-grained feature analysis approach, no finer detail of activity feature or characteristic was computed from raw sensor data (e.g., motion trajectory and wake-up time series based on motion data). In fine-grained feature analysis approach, finer details of activity features or characteristics were computed from raw sensor data (e.g., walking speed, distance covered from motion data, time spent in cooking, and sleep duration). It is observed that both the classes of analysis yielded comparable results associated with early detection process.

Most studies, especially home-based monitoring, did not report any acceptability issues from the study participants. This could be due to the nature of unobtrusiveness of sensors deployed in these studies.

From the perspective of multisite experiments/trials, 7 studies [22–26] [28, 30], reported conducting experiments in multiple sites (smart home residences in case of real-life monitoring or smart home test lab in case of one-time scripted task execution). Among these studies, only in study [28], intersite validation of sensor data, was examined through a statistical method (ANOVA), and other studies did not report any such validation of data gathered in the multisite environment. Interdataset variability can exist from multisite experiments possibly due to selection of sensors mapped to monitoring of certain activities and layout of home or lab settings where subject's routines will be monitored, etc. To overcome this variability, a number of key design considerations should be followed in multisite studies involving sensors. Thus, it is important to standardize the intersite study protocol and that will include selection and placement of sensors, proper sequence of data collection, and planning for data integration. This standardization will enable an effective integrative analysis in which multisite sensor data will be combined, preprocessed, and modeled for better outcomes.

In order to assure the study can produce consistent results/outcomes over the time, the experiments need to consider test-retest reliability design. None of the studies reviewed reported any such test-retest design. Test-retest reliability design can help the studies involving sensor-based activity monitoring in many ways such as (a) selection of relevant features/measures that prove reliability as well as generalizability in measuring older adult's activity, (b) determine the reliable cohort of subjects for further longitudinal monitoring, and (c) determine the reliable duration for monitoring.

The second aim of this review is to present the current state-of-the-art on machine learning methods in predicting cognitive decline/MCI using non-wearable sensor data. From the studies reviewed, it is evident that a wide variety of machine learning techniques were employed in prediction (Tables 1 and 2). Among all the machine learning techniques employed across these studies, Support Vector Machine (SVM) and Random Forest (RF) were the most commonly employed techniques (5 studies). Next to these techniques, the most widely used was Naïve Bayes (NB) (3 studies). After synthesizing machine learning-based analytical approaches from all these studies, main findings are summarized as follows: (1) not all studies specified accuracy of their findings with respect to classifying participants into target groups or predicting MCI diagnosis variables and thus making it difficult to understand the efficacy of their methods and outcomes; (2) the overdependence on few public datasets (CASAS and ORCATECH), (3) class imbalance issue in majority of these studies due to participant sample not representing right proportion between cognitively healthy and MCI population, and (4) heterogeneity in data preprocessing approaches, activity features used, and grain of activity analysis.

Majority of the studies addressed the prediction as the classification problem. In two studies [22, 33], both regression and classification problems were included. One study [28] included regression analysis alone. In the classification analysis, target classes were not consistent across these studies, and they differentiated participants based on either cognitive condition (e.g., cognitively healthy vs. MCI and cognitively healthy vs. dementia) or activity pattern (e.g., normal vs. abnormal behavior). In the regression analysis, some of the neuropsychological test scores or activity performance scores were predicted based on sensor captured activity data. Not all studies specified the accuracy of their findings with respect to abovementioned classification or regression analysis and thus limiting our ability to understand the efficacy of their methods and outcomes.  Table 4 presents the summary of performance metrics corresponding to the best performed machine learning technique reported in each study. In those studies where model metrics were reported for classifying between cognitively healthy and MCI/dementia population (Table 4), we found reasonable level of AUROC metric (Area Under Receiver Operating Curve–degree or measure of separability). However, from the results of best performing classification models (Table 4), there is no evidence found for the classification between MCI and dementia population. Although the classification performances (cognitively healthy vs. MCI/dementia) reported are at reasonably acceptable levels, a variation in these values can be seen. This observation indicates that the research in use of ML methods in this field is still maturing before these methods can be integrated in routine clinical use. As mentioned earlier, 9 out of 12 studies reviewed had utilized public datasets (CASAS and ORCATECH). The overdependence on specific datasets could limit the ability of modeling the behavior/activity of diversified older adults and further could pose generalizability issues with respect to non-US geography settings. In many of the studies, the participant sample did not consist of right proportion from cognitively healthy, MCI and AD population and thus leading to class imbalance issue for the machine learning models. Some of the studies addressed the imbalanced dataset issues through oversampling of minority classes or undersampling of majority classes or ensemble methods in order to avoid the risk of bias in prediction results. Given the heterogeneity in data preprocessing approaches, activity features used, and grain of activity analysis, care should be taken when interpreting the reported results.

Evaluation of machine learning models in all these studies was performed on the internally generated data in respective study, and most of the studies reported either a *k*-fold cross-validation or leave one subject out validation. None of the studies reported the use of any external dataset for evaluating the machine learning model that was trained with internally generated dataset. It will be worthwhile to adopt a two-stage study in evaluating the model and improving the outcomes. Firstly, develop and train the analytical models in one environment/cohort, and secondly, apply these models in another environment/cohort. (beyond cross-validation). An example will be, develop the model in a particular geography set up and deploy and validate in another geography set up.

For the machine learning-based prediction problem of cognitive decline using daily life activities, the critical success factors are appropriate and accurate activity recognition and feature extraction. In this context, traditional machine learning approach shows a heavy dependency on expert knowledge resulting in hand crafted features. There are modern artificial neural network-based methods, such as Convolutional Neural Networks (CNNs), that can automatically learn features (i.e., feature selection/extraction) from input signals without requiring hand crafted features. These deep (multilayered) learning models determine most contributing features and utilize them for successful predictions. But one downside with these deep learning models is that they require a large volume of data to train the models. Only two [29, 31] of the twelve studies reviewed had included deep learning models to classify the inputs, and this indicates research is still emerging as to the use of deep learning models in non-wearable sensor-based early detection of cognitive decline. In these two studies, the results showed that deep learning models outperformed competing traditional ML models in terms of accuracy, precision, and recall.

## 5. Limitations

From the studies reviewed, there have been some limitations observed, and few of them were noted in above sections. To recap, few of the studies provided either limited information or no clarity on mean age of participants/duration of activity monitoring. Besides this aspect, few studies did not specify clearly about participant recruitment strategy, especially consideration of any preexisting/comorbid conditions that could have direct influence on participant's functional performance. While several studies explained clearly about the steps and algorithms used to process sensor data and detect anomalous patterns, others did not provide enough information about how sensor data was preprocessed such as fill-in missed sensor values, activity recognition, and feature extraction and thus hampering reproducibility. Sometimes, the sensor details such as types of sensors deployed, layout, or topology used in smart homes were not completely described and thus limiting the interpretation. Several aspects were not explained in these studies, including sensor selection criteria (e.g., accuracy of measurements, energy efficiency, cost, and maintainability), computational efficiency of machine learning algorithms (e.g., training time and use of computing resources), and among others.

## 6. Conclusions and Future Research Directions

This review covered 12 studies which had the goal of machine learning-based early detection of mild cognitive impairment using smart sensor captured activity data of older adults. For the scope of this review, a count of 12 studies indicates this area of research is still emerging. We found a diverse selection of aspects such as sensors, activity domains, activity features, methods to recognize activity patterns, and detect abnormality leading to the prediction of possible cognitive decline. However, there is no conclusive evidence on superiority of one or more of these aspects over the others, especially on the activity feature (e.g., motion trajectory, sleep pattern, and walking speed) that would be the best indicator of cognitive decline. Nevertheless, the constant publishing of articles shows the growing interest to explore non-wearable sensors in early detection of MCI/AD. Technology community in this research area aims primarily for algorithm novelty, inspired largely by computer vision and machine learning, but the clinical world requires reliable, validated methods for early diagnosis, that are better than traditional methods. All the studies reviewed demonstrate technological developments in this field and applicability for clinical practice as a screening method; however, they all suggest it is far in the future that it becomes an effective diagnostic tool in real-life clinical practice.

As noted earlier, AD is a degeneration that progresses over time, and it is important for researchers to have access to continuously monitored individual's behavior trend data. This longitudinally observed data helps to detect the intraindividual behavioral changes occurred over time and is essential for researchers to develop algorithms and models using longitudinal analysis methods including machine learning and deep learning techniques. Based on this review, we find only a very few openly available datasets that provide this long-term behavioral trend along with incidents of cognitive decline. This is an ongoing challenge in this research field. Thus, we emphasize the need of openly available larger datasets that contain long-term sensor-monitored activity data along with clinically assessed cognitive status. This will motivate researchers leading to many advancements in this field.

In considering the findings from this review, the following recommendations for future research can be made:

- A balanced mix of participants (CH, MCI, AD) that are representative of the target population to which the researcher wishes to generalize the study results so that the risk of bias and concerns regarding applicability to clinical practice can be avoided
- Duration of monitoring long enough to observe the natural evolution of cognitive decline and harness the temporal nature of this degeneration
- Consider the emerging techniques such as deep learning models since they perform better than traditional ML models and eliminate the need of complex and manual feature extraction process. Since deep learning models suffer from computational complexities, research should determine such optimal design that show higher efficiency in resource constrained real life situations
- Finally, selection of sensors and layout in smart homes should be simple, cost effective, generalizable, and reproducible

## Figures and Tables

**Table 1:** General characteristics of the studies included in group 1.

Study	Study design; main objective	Participants; mean age; mean data collection duration	Activities in focus	Machine learning technique	Nature of data gathered/preprocessed	Artifacts detection/correction in sensor data gathered	Approach to address variability, reliability, etc.	Main results
Alberdi et al. [22]	(i) Longitudinal study (ii) Analyze the correlation between activity features and cognitive/behavioral health assessment scores (iii) Detect cognitive/behavioral symptoms that could be indicator of AD	Participants: CH: 13; at risk: 10; MCI: 6; mean age: 84.35; duration: 19.95 months	bADL, iADL, sleep, other daily routines	Regression: SVR, LR, KNN; classification: SVM, AdaBoost, MLP, RF	From sensor event data stream, the activities were recognized, and subsequently activity/behavioral features were computed at day level, and they formed a “time series” data. From this “time series” data, summary statistical features were computed using a sliding window approach for further analysis.	Gaussian detrending—to remove the effect of nonstationary components (e.g., periodic components) in the time series	Reliable change index (RCI) (standardization method) was computed to address intersubject variability in health assessment scores. RCI computation of the test scores in this study utilized test-retest reliability and standard deviation values that the tests have shown in their development cohorts and/or in previous work.	Sensor-based activity observations such as sleep and overnight patterns along with daily routine features contributed significantly to the prediction of various cognitive assessment scores.
Schinle et al. [23]	(i) Longitudinal study (ii) Detect possible indicators of onset of dementia using individuals' day-night rhythm and night-time activity as relevant parameters	Participants: 10; mean age: NS; duration: 13 months	Movement patterns to infer sleep patterns	Local outlier factor (clustering)	From the raw sensor events data stream, two types of events were derived, namely, motion events and outside home events. Based on density of these events, three measures such as wake-up time, bed time, and night time activity count were determined. Each of these measures formed a time series data for learning behavioral profile and trend.	Nothing specific was discussed in terms of artifact detection and correction. However, authors noted that the single motion sensor could not detect many activities outside of sensor's vision (e.g., latent motion) and that would lead to the understanding of “no movements” impacting the accuracy of prediction.	NS	From the activity trend data, the wake-up times/bed times were recognized, classified as no anomaly or slight anomaly or severe anomaly, based on anomaly detection rules defined. These rules were defined heuristically after examining the distribution of the wake-up and bed times for several households.
Sharma et al. [24]	(i) Longitudinal study (ii) Detect early symptoms of MCI using ADL data from sensors (iii) Address to fill in data gaps caused by sensor failures	Participants: 50; mean age: NS; duration: 6 months	Daily routine pattern	RNN	Expressed human routine as a time series inference based on the raw data stream from sensors.	Data gaps (missing sensor values) caused due to faulty sensors/dead sensors were filled in using time series prediction techniques such as RNN. Missing sensor values indicate the presence of certain noise in the incoming sensor data stream.	NS	All the activities recognized from sensor data on a particular day of a participant were compared against the same participant's data from previous day to compute the deviation; if this deviation was noted for more than month, abnormality was detected and referred for further clinical evaluation.
Akl et al. [25]	(i) Longitudinal study (ii) Home-based automatic detection of MCI symptoms through individual's room activity distributions	Participants: CH-59; a-MCI: 11; na-MCI: 15; mean age: not clear; duration: 3 years average	General home activity patterns	Affinity propagation (clustering)	Activity data from raw sensor data stream was used to compute room activity probability distributions. These probability distributions were considered as discretized values over a fixed time interval but not treated as a time series data.	Discarded sensor readings of certain days when unusual activity patterns (zero, too many, etc.) were seen and those could be due to variety of reasons such as study participant had visitors or some sensor failed, etc.	NS	Individual's activity distributions, combined from all the four rooms, namely, bedroom, bathroom, living room, and kitchen were found to be significant contributor in predicting an individual as “Cognitively intact” or “MCI.” Within MCI class, individuals transitioned to subtype “a-MCI” showed significant changes in their bedroom activity distributions that were mainly attributed to disturbed sleep patterns.
Akl et al. [26]	(i) Longitudinal study (ii) Autonomous detection of MCI symptoms based on walking speed and general activity recognized through unobtrusive sensing technology	Participants: CH: 79; MCI: 18; mean age: not clear (70 and above); duration: 171.9 weeks	bADL, other daily routines	SVM, RF	From the raw sensor events data stream, walking-related predefined measures were computed, and these measures were transformed into features using signal processing approach (based on sliding window). These features served as data points for machine learning modeling and prediction.	Discarded sensor readings of certain days when unusual activity patterns (zero, too many, etc.) were seen and those could be due to variety of reasons such as study participant had visitors or some sensor failed, etc.	NS	Trajectories of weekly walking speed-related measures and the participant's age and gender were the most important for detecting MCI in older adults. The feature, “trajectories of measures,” refers to the concatenation of corresponding measures as they appeared in each window (“l” week).
Albeiruti et al. [27]	(i) Longitudinal study (ii) Detect sudden and gradual abnormalities in behavior of older adults based on movement pattern recognized through simple motion sensors	Participants: CH: 1; mean age: NS; duration: 219 days	Movement patterns	HMM	Sensor firing states (from raw sensor data) were treated as observed states in learning the Hidden Markov model's parameters which characterized the subject's behavior. Probability distributions of a sensor firing after another sensor were derived from observed data (training data).	A manual cleanup was done to discard the sensor readings where an “ON” state was observed without having a corresponding “OFF” state.	NS	The behavior model (movement pattern based) was able to detect abnormalities on specific days within the monitoring period. Abnormal days could be indicator of cognitive decline.

ADL: activities of daily living; a-MCI: amnestic MCI; bADL: basic ADL; CH: cognitively healthy; HMM: Hidden Markov Model; MCI: mild cognitive impairment; na-MCI: nonamnestic MCI; NS: not specified; RF: Random Forest; RNN: recurrent neural network; SVM: Support Vector Machine.

**Table 2:** General characteristics of the studies included in group 2.

Study	Study design; main objective	Participants; mean age; mean data collection duration	Activities in focus	Machine learning technique	Nature of data gathered/preprocessed	Artifacts detection/correction in sensor data gathered	Approach to address variability, reliability, etc.	Main results
Lussier et al. [28]	(i) Cross-sectional study (ii) Examine how strongly the sensor-based activity data was related to the MCI clinical diagnosis, cognitive performance, and performance-based measures	Participants: CH: 26; MCI: 22; mean age: NS; duration: NS	bADL, iADL	Regression analysis	Sensor readings were used to calculate the duration of the scripted tasks performed by the participants. Single trial at two different sites was reported, and there was no relevance of a time series treatment of measures derived from sensor data.	NS	Intersite validation of sensor data was examined through ANOVA: both sites were comparable in terms of time spent in each living area as well as for the use of domestic appliances and storage.	Sensor-based activity data were associated with memory and executive performances and significantly contributed to the prediction of MCI.
Arifoglu et al. [29]	(i) Longitudinal and cross-sectional study (ii) Recognize activity patterns from sensor-based activity data and detect abnormal behavior related to dementia	Longitudinal study participants: CH: 1; cross-sectional study participants: CH: 20; mean age: NS; duration: not clear	bADL, iADL, sleep	CNN, LSTM	Sensor data is discretized by sliding window approach with a constant time-slice length of 60 sec and forming a time series chunk of tXf matrix where rows are time slices and columns are sensor readings. This preprocessed data was given as input for CNN for classification.	NS	Cohen's Kappa statistics was computed in order to show the robustness of the proposed CNN-2D classifier. A value of 0.64431 indicated a substantial agreement.	When both temporal and feature dimensions were considered, results of activity recognition and anomalous pattern detection were found to be promising, especially in case of repetition-related activities and confusion-related activities.
Paudel et al. [30]	(i) Longitudinal study (ii) From the sensor-based activity data, derive behavioral/activity features and determine any anomalous activity patters that could be predictive of onset of dementia	Participants: CH: 5; MCI: 5; age: 80-91 years; duration: not clear	bADL, iADL, sleep	Logistic regression, LDA, DT, SVM, KNN, RF, Ada Boosting, one-class SVM	From the raw sensor data stream, features related to each activity were derived and that would serve as data points for modeling. These data points were treated as discrete data points rather than any time series while training and testing the models.	NS	NS	Regardless of any specific activity domain, features derived from all activities contributed significantly to classify the subject into healthy or MCI group.
Gochoo et al. [31]	(i) Longitudinal study (ii) From sensor-based activity data, derive Martino-Saltzman's (MS) travel patterns that could be indicator of individual's cognitive state	Participants: CH: 1; mean age: NS; duration: 625 days	Movement patterns	Deep CNN, NB, GB, RF	Raw dataset representing a long list of consecutive movements (motion sensor data) is segmented into groups of travel episodes. Episode starts when there is any movement is occurred in the raw sensor data after the end of previous episode and the episode stops if there is no motion for more than 10 seconds. Each episode was converted to a 32 × 32 binary image which was input to the DCNN model.	Discarded sensor readings of certain days when unusual activity patterns (zero, too many, etc.) were seen and those could be due to variety of reasons such as study participant had visitors or some sensor failed, etc.	NS	Each episode was classified into one of the patterns—direct, pacing, lapping, and random. These results could be precursor to further clinical evaluation and diagnosis.
Li et al. [32]	(i) Cross-sectional study (ii) Perform a quick screening of older adults and classify into dementia or nondementia group utilizing motion trajectory-based features derived from iADL data	Participants: CH: 72; MCI: 43; dementia: 7; age: 60–74 years; duration: NS	bADL, iADL	Bayesian network, SVM, RF, NB	As the participants performed their activities, the IDs of motion sensors triggered for each activity were combined together to form a motion “Trajectory” of that activity. Appropriate features were extracted from these trajectories to be fed into learning algorithm.	NS	NS	Wandering patterns such as pacing and lapping (as represented trajectory features) were significantly different between subjects with dementia and without dementia. Subjects classified into dementia group could not be differentiated between MCI and CH perhaps due to nature of activities performed in very short span of time.
Dawadi et al. [33]	(i) Cross-sectional study (ii) Automatically calculate the activity performance score from sensor captured activity data; correlate this score with expert assigned as well as predict cognitive health condition based on this automated score	Participants: CH: 145; MCI: 32; Dem: 2; mean age: not clear; multiple age groups- *n* = 37 < 45 yo; *n* = 27 45-59 yo; *n* = 84 60-74 yo; *n* = 31 > 75 yo; duration: 1 hour	bADL, iADL	SVM (bagged), NN, NB	From sensor event data stream, the activities (scripted tasks) were recognized, and subsequently, activity features were computed which would indicate the quality of task completion and quantum of parallelism. Single trial at a single site was reported, and there was no relevance of a time series treatment of measures derived from sensor data.	NS	Two trained neuropsychologists observed the participants performing the tasks and recorded two scores, namely, task accuracy and sequencing score. The sensor-derived task features were examined to be correlated to these observers rated scores. Interrater reliability agreement came out to be 97.88% and 99.57% for the accuracy and sequencing scores, respectively.	The correlation (*r*) between smart home sensor-derived features and task accuracy scores was found to be statistically significant (rather than task sequencing score). While predicting cognitive health, study was able to classify between CH and dementia with a better accuracy than classifying between CH and MCI.

ADL: activities of daily living; a-MCI: amnestic MCI; bADL: basic ADL; CH: cognitively healthy; CNN: Convolutional Neural Network; DT: decision tree; GB: gradient boost; HMM: Hidden Markov Model; iADL: instrumental ADL; KNN: K nearest neighbors; LDA: linear discriminant analysis; LSTM: long short-term memory; MCI: mild cognitive impairment; na-MCI: nonamnestic MCI; NB: Naïve Bayes; NN: neural network; NS: not specified; RF: Random Forest; RNN: recurrent neural network; SVM: Support Vector Machine.

**Table 3:** Characteristics of sensor monitoring method.

Study	Nonwearable sensor and count	Activity domain (sensor monitored)	Activity/routine (sensor monitored)	Other measurements used for analysis (nonsensor data)	Activity recognized?	Anomaly detected?	Fitness for real-world settings	Grain of activity feature analysis
Alberdi et al. [22]	PIR motion sensor: 16 Door sensor: 2 Temperature sensor: 4 Light sensor: 16	Movement	Walking, out of home	Behavior/health assessment: arm curl and TUG mobility test, digit-cancellation test, RBANS and PRMQ, GDS	Yes	Yes	Early in maturity for deployment	Fine-grained feature analysis
		Self-care	Eating, personal hygiene, toileting, and incontinence					
		Domestic life area	Cooking					
		Passive activity	Sleeping, resting					
		Daily routine	Daily routine					
Schinle et al. [23]	PIR motion sensor: 1 Door contact sensor: 1	Movement	Motion, out of home	NA	Yes	Yes	Early in maturity for deployment	Coarse-grained feature analysis
Sharma et al. [24]	PIR motion sensor, temperature sensor, vibration sensor count: NS	Daily routines	Daily routines	NA	No	Yes	Only for initial/quick screening	Coarse-grained feature analysis
Akl et al. [25]	PIR motion sensor: 7 Door contact sensor: 3	Daily routines	Home activity	NA	No	No	Early in maturity for deployment	Coarse-grained feature analysis
Akl et al. [26]	PIR motion sensor: 13 Motion sensor: 4 Door contact sensor: 3	Movement	Walking, out of home	Age, gender	No	No	Early in maturity for deployment	Coarse-grained feature analysis
		Daily routines	Daily routines					
Albeiruti et al. [27]	PIR motion sensor: 31	Movement	Motion	NA	No	Yes	Early in maturity for deployment	Coarse-grained feature analysis
Lussier et al. [28]	*Z*-wave infrared motion detector: 6 Electric sensor: 2 Door contact sensor: 4	Domestic life area	Performing house work, cooking	Executive function and memory assessment score Activity performance score by expert	No	No	Early in maturity; good for initial screening for further follow-up	Fine-grained feature analysis
		Learning, applying knowledge	Reading, attend phone call					
Arifoglu et al. [29]	Motion sensor: 31 Door contact sensor: 3	Movement	Out of home	NA	Yes	Yes	Early in maturity for deployment	Coarse-grained feature analysis
		Self-care	Eating, personal hygiene, toileting					
		Domestic life area	Cooking					
		Passive activity	Sleeping, resting					
Paudel et al. [30]	PIR motion sensor: 23 Door sensor: 6 Temperature sensor: 5 Light sensor: 23	Movement	Out of home	NA	Yes	No	Early in maturity for deployment	Fine-grained feature analysis
		Self-care	Eating, personal hygiene, toileting					
		Domestic life area	Cooking					
		Passive activity	Sleeping, resting					
Gochoo et al. [31]	Motion sensor: 31	Movement	Motion	NA	No	No	Good for initial screening for further follow-up/diagnosis; possibility for detecting wandering movement in real-time application	Coarse-grained feature analysis
Li et al. [32]	Motion sensor: 52	Domestic life area	Performing house work, cooking	Activity performance score by expert	No	No	Early in maturity; good for initial screening for further follow-up	Coarse-grained feature analysis
		Self-care	Taking medicines					
		Learning, applying knowledge	Preparing letters, search for specific video, search for a specific outfit					
		Communication	Attend phone call					
Dawadi et al. [33]	Motion sensors: 27 Door sensors: 10 Item sensors (kitchen): 5 Temperature sensors: 2 Light sensors: 4 Sensors to monitor water and burner use: 3	Domestic life area	Performing house work, cooking	Activity performance score by expert Cognitive diagnosis based on neuro-psychological tests	No	No	Early in maturity for deployment	Fine-grained feature analysis

PIR: passive infrared; RBANS: Repeatable Battery for the Assessment of Neuropsychological Status; GDS: Geriatric Depression Scale; NS: not specified; PRMQ: Prospective and Retrospective Memory Questionnaire; TUG: Timed Up and Go.

**Table 4:** Summary of performance metrics corresponding to best performed machine learning technique reported in each study.

Study	Target	Machine learning technique	Model evaluation including cross-validation method	Classifier metrics	Regression metrics	Key observations	Software used for analysis
Alberdi et al. [22]	Predict the absolute test score using sensor-derived features (regression)	SVR (RBF)	For both regression and classification analysis, 10-fold CV was performed, and models were tested with the internal dataset gathered; no mention of evaluating the model on any external dataset.	NA	Correlation coefficient (*r*): 0.55 MAE: 5	For mobility tests, TUG demonstrated a moderate to strong correlation with sensor-derived behavioral features.	(i) R studio for computing time series statistics (ii) Weka for prediction modeling (correlation and classification analysis)
	Detect a reliable change in health assessment scores; no decline (+ve class) vs. decline (-ve class)	RF (on PCA reduced dataset)		Recall: 28% *F*-score: 0.33 AUC-ROC: 0.65 AUC-PRC: 0.54	NA	Person's improvement/decline in mobility domain detected from sensor derived features indicates early symptoms for MCI.	
Schinle et al. [23]	Normal vs. slight anomaly vs. severe anomaly (activity)	Local outlier factor	NS	Accuracy: 90.9% for wake-up time and 93.2% for bed time	NA	Deviation in any of the learned wake-up time/bed time/night time activity profiles (outlier) indicates the anomaly.	NS
Sharma et al. [24]	Normal vs. abnormal (routine)	RNN	No CV was reported for evaluating the performance of predictor. CV was included in the training process of RNN model that filled in missing values.	NS	NA	Abnormality was detected by using daily routine vector comprising of sensor values. Classifier performance was not explained.	NS
Akl et al. [25]	CIN vs. MCI	Affinity propagation	As a first step, model was trained and tested with this 80 : 20 split; as a second step, in order to find the validity of the model, a leave-one-subject-out CV was performed using only the 22 subjects transitioned to MCI during study period. No mention of any external dataset for evaluation.	*F*_0.5_ score: 0.789	NA	A time frame of 20 weeks was found to be the duration that generates room activity distributions that are most conducive to detecting MCI in older adults.	NS
Akl et al. [26]	MCI vs. CH	SVM (RBF)	Entire dataset was divided into three groups for a 3-fold CV such that each group had approximately the same total number of data points (feature vectors) pertaining to each class (cognitively intact and MCI). No mention of any external dataset for evaluation.	AUC-ROC: 0.97 AUC-PRC: 0.93	NA	Walking speed-related features were more effective in predicting the MCI condition than any other features. Analyzing activity features for a time window of 24 weeks yielded a best performance.	MATLAB
Albeiruti et al. [27]	Normal vs abnormal (behavior trend)	HMM	Initial behavioral model was created with the entire dataset. Later, the resulting model was used as a standard model to be compared with the same dataset days one by one. There is no clarity as to what were the ground truth in evaluating the outcomes of this approach.	NS	NA	Person's movement transitions from one location to another location were used for behavior modeling. Classifier performance was not explained.	MATLAB
Lussier et al. [28]	Predict the MCI diagnosis variable using the sensor-based iADL measures and expert rated performance scores (regression)	Regression analysis	NS	NA	*R* ^2^: 0.47 *F*: 22.01 (sig < 0.001)	Sensor-based iADL measures represent time spent in activities related to mobility, hygiene, and cooking	IBM SPSS
Arifoglu et al. [29]	Abnormal vs. normal behavior	LSTM	Entire dataset was divided into 3 partitions one each for training, validation, and testing by fixed number of days, i.e., 139 days : 15 days : 70 days on 224 days of monitoring. No mention of any external dataset for evaluation.	Sensitivity: 98.67% Specificity: 75.48%	NA	Study results report that LSTM are more suitable to detect repetition and order-related abnormal activities since it can relate current input with the upcoming ones.	Keras Deep Learning library's and Theano's implementation of CNNs and LSTM
Paudel et al. [30]	CH vs. MCI	RF	A 10-fold CV was performed with the internal dataset gathered; no mention of any external dataset for model evaluation.	Accuracy: 73% Precision: 73% Sensitivity: 73% Specificity: 73% *F*-score: 0.73 AUC-ROC: 0.72	NA	—	Python using the sci-kit learning tool
Gochoo et al. [31]	Direct vs. pacing vs. lapping vs. random (travel pattern)	Deep CNN	A 10-fold CV was performed with the internal dataset gathered; no mention of any other external dataset for model testing/evaluation.	Accuracy: 97.84% Precision: 97.9% Sensitivity: 97.8% Specificity: 99.3% *F*-score: 0.978	NA	—	NS
Li et al. [32]	Dementia vs. nondementia (nondementia includes CH and MCI)	Bayesian network	Leave-one-out CV was performed with the internal dataset gathered; no mention of any other external dataset for model testing/evaluation.	Precision: 98.3% Sensitivity: 98.3% AUC-ROC: 0.851	NA	The basis of this study is that the moving trajectories of the older adults with dementia are different from those of without dementia.	NS
Dawadi et al. [33]	Map the sensor-derived activity features to the direct observation scores (regression)	SVM regression (with bagging)	NS	NA	Correlation coefficient (*r*): 0.58	The correlation (*r*) between smart home sensor derived features and task accuracy scores was found to be statistically significant.	
	MCI vs. CH	SVM (with cost sensitive learning)	Leave-one-out CV with internal dataset gathered; no mention of any external dataset for evaluation.	*F*-score (class A): 0.37 *F*-score (class B): 0.78	NA	Classification performance was not strong due to the individuals in these two groups do have quite a bit of overlap in functional performance (activities).	
	Dementia vs. CH	SVM	Leave-one-out CV with internal dataset gathered; no mention of any external dataset for evaluation.	*F*-score (class A): 0.93 *F*-score (class B): 0.99	NA	Classification performance was best because the individuals in these two groups exhibited a vast difference in performing the scripted tasks/activities.	

AUC: area under the curve; CH: cognitively healthy; CIN: cognitively intact; CNN: Convolutional Neural Network; CV: cross-validation; HMM: Hidden Markov Model; iADL: instrumental activities of daily living; LSTM: long short-term memory; MAE: mean absolute error; MCI: mild cognitive impairment; NA: not applicable; NS: not specified; PCA: principal component analysis; PRC: precision-recall curve; RBF: radial basis function; RF: Random Forest; ROC: receiver operating characteristic curve; SVM: Support Vector Machine; SVR: Support Vector Regression; TUG: Timed Up and Go.

## Data Availability

None.
